# Digital ischaemia of the upper limbs in middle age: consider arterial thoracic outlet syndrome until proven otherwise!

**DOI:** 10.1093/rap/rkaa045

**Published:** 2020-12-01

**Authors:** Wissam Al-Jundi, WooSup Michael Park

**Affiliations:** 1 Norwich Vascular Unit, Norfolk and Norwich University Hospital NHS Trust, Norwich, UK; 2 Heart and Vascular Institute, Cleveland Clinic Abu-Dhabi, Abu-Dhabi, United Arab Emirates

**Keywords:** digital ischaemia, arterial thoracic outlet syndrome, cervical rib

## Abstract

**Objectives:**

Patients presenting with digital upper limb ischaemia are occasionally referred to rheumatology services to rule out vasculitis. We aimed to present two cases of delayed diagnosis of arterial thoracic outlet syndrome (aTOS) in middle-aged patients presenting with digital ischaemia in order to raise awareness of this important pathology that requires timely surgical intervention.

**Methods:**

Two cases of progressive ischaemia of the right upper extremity caused by primarily undiagnosed compression of the subclavian artery by an accessory cervical rib are presented. The case notes, radiological images, intra-operative and postoperative findings for both patients were reviewed. Patients were followed up after ≥6 months to assess prognosis.

**Results:**

Both patients had a working diagnosis of Buerger’s disease and had been treated with prostaglandin infusions before establishment of the diagnosis of arterial thoracic outlet syndrome. Both patients were heavy smokers, and one patient had bilateral symptoms and a history of axial SpA and positive HLA-B27. Late presentation in one patient led to the loss of three fingers and the need for plastic reconstructive surgery after cervical rib resection and revascularization. In the other patient, surgical thrombectomy of the upper limb arteries along with resection of a cervical rib and repair of the subclavian artery with an interposition graft were necessitated to heal digital ulcers successfully.

**Conclusion:**

A high index of suspicion of aTOS should be maintained in middle-aged patients presenting with digital or upper limb ischaemia even in the presence bilateral symptoms or relevant risk factors of other diagnoses, such as smoking or a positive rheumatological history.

Key messages

- Arterial thoracic outlet syndrome should be considered in all middle-aged patients presenting with upper limb ischaemia.
- Smoking could be a risk factor that precipitates symptoms with arterial thoracic outlet syndrome because it aggravates thrombosis.
- Arterial thoracic outlet syndrome can be a surgical emergency, and timely referral is paramount.

## Introduction

Digital ischaemia of the upper limbs remains a challenging presentation for clinicians owing to the long list of differential diagnoses that are covered in practice by different specialties. Thoracic outlet syndrome (TOS) occurs because of compression of the brachial plexus (nTOS), the subclavian vein (vTOS) or the subclavian artery (aTOS). Owing to its rarity, aTOS is occasionally undiagnosed. Symptomatic compression of the subclavian artery represents only 1% of TOS variations and is almost always caused by bony abnormality, such as an anomalous first rib or a cervical rib [1]. The latter has an incidence of 0.74% in the general population [2] and can compress the subclavian artery between the first rib and the anterior scalene muscle, resulting in stenosis and intimal injury, with post-stenotic dilatation, thrombosis and distal embolization. Indeed, digital ischaemia attributable to microembolization is the most common presentation of aTOS. However, this presentation is also common with other causes of digital ischaemia, such as proximal sources of embolization, vasculitis, thromboangitis obliterans, CTDs and atherosclerotic disease of the upper limbs.

The aim of this paper is to present two cases of delayed diagnosis of aTOS. Exclusion of this pathology is paramount, because timely surgical intervention can significantly reduce morbidity.

## Case 1

A 43-year-old right-hand-dominant male patient, with a history of axial SpA, bilateral sacroiliitis with positive HLA-B27 and possible history of uveitis, was referred in January 2020 with bilateral digital ischaemia, worse on the right side. Six weeks before that, he presented under the rheumatology services with a milder degree of skin changes affecting his fingers in the form of blisters. His working diagnosis was Beurger’s disease, in the context of a history of heavy smoking. He was advised to stop smoking and received a prescription for glyceryl trinitrate patches and calcium channel blocker tablets, along with arrangements of outpatient prostaglandin infusion sessions. At that time, the patient was seen in the outpatient department; he declined to wait for vascular assessment and subsequently did not attend a magnetic resonance angiogram appointment. He reported gradual progression of ischaemia over the course of 6 weeks despite vasodilator infusions and medical treatment and was referred to the oncall vascular team. He reported no previous history of arm claudication, cardiovascular disease or exposure to vibration tools. He was unemployed and used to be a van driver.

**Fig. 1:**
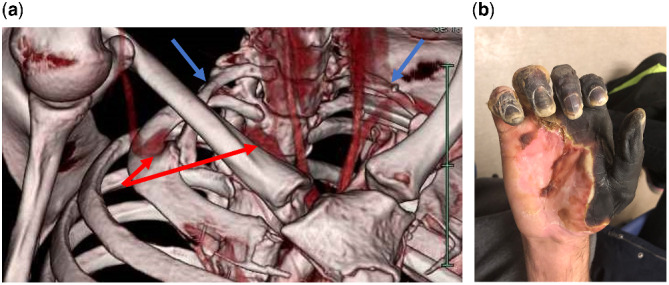
Radiological and clinical findings of Case 1 (**A**) CT angiogram of the right upper limb showing bilateral cervical ribs (blue arrows) and compression of the right subclavian artery in the abduction position (red arrows). The right subclavian artery was found to be patent in the neutral position, with mild dilatation distal to the cervical rib. (**B**) Dry necrosis of the right hand 6 weeks postoperatively. The old scar on the volar side of the wrist from penetrating trauma at age of 14 years is demonstrated.

On examination, it was noticed that there was difficulty in recording blood pressure in the right arm, with a profoundly ischaemic hand in the form of fixed mottling of the thumb, index and middle fingers, whereas the ulnar side was pale, insensate and with reduced motor function. He was noticed to have a scar on the volar aspect of his wrist and thenar eminence from previous laceration at the age of 14 years, which necessitated reconstructive surgery in a different hospital. He had an easily palpable subclavian pulse with suspected dilatation, but no pulses below. He reported improvement in the left-hand blisters, and the left side had a normal full complement of pulses. All serological markers of vasculitis were negative. An urgent CT angiogram revealed the presence of bilateral cervical ribs, compression of the right subclavian artery in the abduction position and occlusion of the right brachial artery owing to thrombosis, with normal upper limb circulation on the left side (Fig. 1A).

Hence, he underwent an emergency right cervical rib resection and brachial thrombectomy. The latter was successful in restoring pulsatile flow to the elbow level, but attempts at smaller vessel thrombectomy were not successful owing to the presence of chronic organized thrombus. The patient had an uneventful recovery, with a palpable brachial pulse and restoration of monophasic Doppler signal in the ulnar artery at the wrist level. The irreversibly ischaemic radial side of his hand became fully demarcated dry necrosis, whilst the ulnar side was preserved (Fig. 1B). He also underwent left cervical rib resection 6 weeks later and is currently awaiting an attempt at reconstructive surgery of his right hand using a groin flap.

## Case 2

**Fig. 2:**
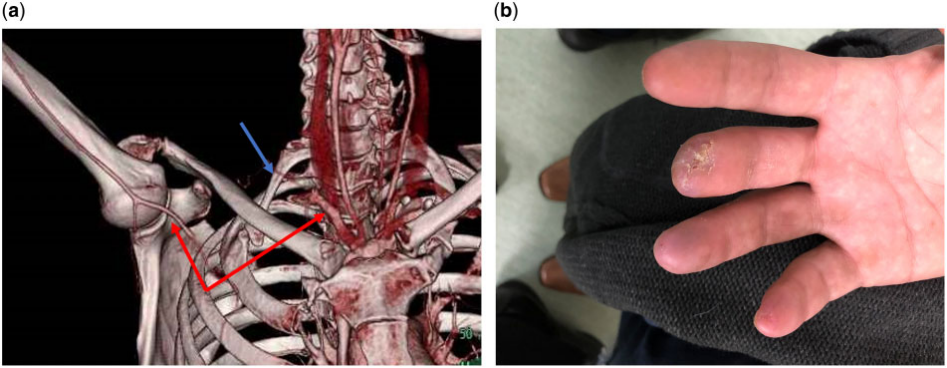
Radiological and clinical findings of Case 2 (**A**) CT angiogram of the right upper limb showing unilateral right cervical rib (blue arrow) and compression of the right subclavian artery in the abduction position (red arrows). (**B**) Right hand 6 months postoperatively, with healed terminalization of the middle finger.

A 39-year-old right-hand-dominant male patient who is normally fit and well presented in July 2019 with right hand resting pain and digital wet necrosis of the tip of his middle finger. He presented 2 months before that with a milder degree of symptoms and received a diagnosis of Buerger’s disease, based on his heavy smoking history, absence of distal pulses below the brachial artery and CT angiogram evidence of absent runoff in small vessels below the elbow level. He was treated with anticoagulation and prostaglandin infusion. However, when he presented with worsening symptoms 2 months later, it was noticed that he had lost his subclavian and brachial pulses. A repeat CT angiogram revealed thrombosis of an aneurysmal right subclavian artery and the presence of a unilateral right cervical rib that was inadvertently unreported on his previous CT scan (Fig. 2A).

The patient underwent emergency right cervical rib resection and subclavian artery interposition bypass with a reinforced 8 mm Dacron graft. Brachial embolectomy retrieved organized brachial thrombus but, as for Case 1, was unsuccessful in retrieving old clots in the small vessels of his forearm. Postoperatively, the patient had a well-perfused hand, palpable brachial pulse and excellent Doppler signals at the digital level. He underwent debridement of his fingers after 6 weeks, and follow-up after 6 months showed healed fingers and a patent bypass graft (Fig. 2B).

## Discussion

Thoracic outlet syndrome is defined as a constellation of clinical disorders attributable to compression of the brachial plexus and/or the subclavian vessels at their thoracic outlet [3, 4]. Two cases of delayed diagnosis of aTOS are described. Both patients presented with digital ischaemia, which is the commonest presentation of aTOS that tends to affect young and active adults. The mean age in most published series is 37 years, with a similar proportion of men and women reported [5–7]. Bony abnormality is usually the culprit, with cervical ribs being most common. Less common causes of aTOS include anomalous ﬁrst ribs, ﬁbrocartilaginous bands associated with the anterior scalene muscle, and hypertrophic callus from healed clavicle or ﬁrst rib fractures [8, 9]. As a result of repeated compression, subclavian artery dilatation usually occurs, and turbulent blood flow results in formation of perimural thrombi that can detach and migrate as embolic material to the upper limb arteries. Although both patients presented with digital ischaemia of variable degree, the ischaemia in Case 1 was compounded by a history of penetrating injury to his right wrist that necessitated reconstructive surgery with reported arterial injury, which meant that he had compromised collateral reserve in the distal forearm and palmar arch.

Thorough clinical and radiological assessments are paramount for detecting patients with aTOS. The former includes measurement of blood pressure in both upper limbs, assessment of the upper limb pulses, palpation for cervical ribs and performance of compression manoeuvres, such as Adson and Roos tests. An initial radiological assessment that can be arranged by rheumatologists is a chest x-ray including cervical spine views, which often demonstrates the offending bone abnormality, whereas an MRI has the advantage of occasionally demonstrating fibrous bands. Vascular specialists frequently request duplex US, which can demonstrate aneurysmal changes or elevated ﬂow velocities correlated with a compressive stenosis. Imaging protocols usually involve the patient’s arm being positioned in abduction to demonstrate the compression. However, this should be accompanied by imaging in the neutral position because the abduction position can give a false impression of an occluded subclavian artery when it is simply compressed. In addition, compression of the subclavian artery was found in 10% of healthy, asymptomatic young individuals with abduction of the arm at the shoulder joint [10]. In emergency presentations, a CT angiogram is the most readily available modality and can provide anatomical details regarding the presence of an osseous abnormality, a subclavian artery post-stenotic dilatation, the extent and the location of thromboembolic occlusions.

Owing to the common presence of a bony compression, treatment of aTOS almost always requires surgical intervention in the form of resection of the offending cervical rib. In asymptomatic patients, controversy exists over prophylactic resection of cervical ribs. However, we believe that the potential catastrophic consequences of conversion to symptomatic aTOS and the common association with nTOS justify surgical resection. For patients presenting with complications owing to thrombosis or aneurysmal degeneration of the subclavian artery, bony resection should be accompanied by surgical reconstruction of the subclavian artery. The latter was needed in Case 2 in the form of a subclavian artery interposition bypass, whereas Case 1 had a patent subclavian artery with mild post-stenotic dilatation. Despite persistent occlusion of the small vessels, the presence of sufficient collateralization promoted healing of the digital ischaemia in the second patient and minimized the extent of amputation in the first patient.

The presented cases highlight several challenges in making the diagnosis of aTOS. It remains a rare condition with several other, more common pathologies that can cause hand ischaemia. Embolization can originate from more proximal sources, such as a cardioembolic origin. This tends to be more common in older patients with a history of arrythmias, hence physical examination and ECG are relevant investigations in this context. Atherosclerotic peripheral arterial disease is another differential in this age group, but this tends to have a more insidious onset, and patients present with arm claudication before progression to ulceration of their digits. In younger patients, vasculitis, CTDs, vibration syndromes and aTOS are likely causes of digital ischaemia. Our patients were heavy smokers, which raised suspicion of thromboangitis obliterans (Buerger’s disease). Case 1 had history of sacroiliitis, possible uveitis, raised HLA-B27 and bilateral symptoms; hence, a systemic cause was suspected initially. Low index of suspicion, relatively delayed imaging studies and underreporting of the subclavian artery abnormality and cervical ribs contributed to the delay in diagnosing aTOS. Misdiagnosis of aTOS has been reported before, with patients labelled as having Buerger’s disease [10–12]. We believe that the presence of a smoking history can be a factor in causing symptomatic presentation of aTOS owing to the increased risk of thrombosis, whereas Buerger’s disease should be a diagnosis of exclusion. The presence of bilateral symptoms favours a systemic aetiology, but aTOS should not be ruled out, because cervical ribs tend to occur bilaterally in 50% of cases [13]. Both cases also lacked the typical angiographic appearance of corkscrew-like collateral blood vessels that characterizes Buerger’s disease. Other clinical features of Buerger’s disease include superficial phlebitis or RP, which can be present in ∼40% of patients [14]. In contrast, the lack of occupational exposure to vibrations in both patients rendered vibration-induced disease an unlikely explanation.

*Funding*: No specific funding was received from any bodies in the public, commercial or not-for-profit sectors to carry out the work described in this article.

*Conflict of interest*: The authors have declared no conflicts of interest.
